# Characterization of Gas–Liquid Two-Phase Slug Flow Using Distributed Acoustic Sensing in Horizontal Pipes

**DOI:** 10.3390/s24113402

**Published:** 2024-05-25

**Authors:** Sharifah Ali, Ge Jin, Yilin Fan

**Affiliations:** 1 Petroleum Engineering Department, Colorado School of Mines, Golden, CO 80401, USA; ssali@mines.edu; 2 Geophysics Department, Colorado School of Mines, Golden, CO 80401, USA; gjin@mines.edu

**Keywords:** distributed acoustic sensing, distributed fiber-optic sensing, slug flow characterization, multiphase flow sensors, gas–liquid slug flow, horizontal pipe flow, flow monitoring, multiphase flow meter

## Abstract

This article discusses the use of distributed acoustic sensing (DAS) for monitoring gas–liquid two-phase slug flow in horizontal pipes, using standard telecommunication fiber optics connected to a DAS integrator for data acquisition. The experiments were performed in a 14 m long, 5 cm diameter transparent PVC pipe with a fiber cable helically wrapped around the pipe. Using mineral oil and compressed air, the system captured various flow rates and gas–oil ratios. New algorithms were developed to characterize slug flow using DAS data, including slug frequency, translational velocity, and the lengths of slug body, slug unit, and the liquid film region that had never been discussed previously. This study employed a high-speed camera next to the fiber cable sensing section for validation purposes and achieved a good correlation among the measurements under all conditions tested. Compared to traditional multiphase flow sensors, this technology is non-intrusive and offers continuous, real-time measurement across long distances and in harsh environments, such as subsurface or downhole conditions. It is cost-effective, particularly where multiple measurement points are required. Characterizing slug flow in real time is crucial to many industries that suffer slug-flow-related issues. This research demonstrated the DAS’s potential to characterize slug flow quantitively. It will offer the industry a more optimal solution for facility design and operation and ensure safer operational practices.

## 1. Introduction

Multiphase flow in pipes is a common but also complex phenomenon occurring in various fields, such as petroleum engineering, geothermal applications, nuclear engineering, etc. For example, in the petroleum industry, oil and gas flow is inevitable due to the pressure reduction and temperature variations along the wellbore and pipeline systems. As energy demand increases and the world explores deeper harsh zones to increase oil and gas recovery, multiphase-flow-related safety issues are becoming more complex and concerning. Critical parameter monitoring in real time is essential for better production management and optimization. Detecting and forecasting the circumstances of the well at earlier periods have a substantial effect on well control strategies; health, safety, and environment (HSE); and risk management [1]. This will give the operators the ability to take the appropriate action at the right time [2].

There are several types of point sensors that can characterize slug flow, such as γ-ray sensors, wire-mesh sensors, electrical impedance (capacitance and conductance) sensors, microwave sensors, optical sensors, etc. They are either not safe (γ-ray sensors have a radioactive source), intrusive (such as the wire-mesh sensors), or have special requirements on the type of fluids or flow pattern (see details in [3]). One significant drawback is that they all require installation directly into the piping system, necessitating the cutting of pipes and their subsequent connections which adds another failure factor. Moreover, these technologies can only provide point measurements at a specific location where the sensor is installed. Considering the cost of each sensor, it is not economically feasible to install one for each individual pipeline/well that requires detailed monitoring.

The fiber-optic sensing technology is developing rapidly and signifies the future of multiphase flow supervising and monitoring. Fiber optics can prevail over the limitations of conventional electrical sensor arrays due to their smaller size, non-intrusive features, corrosion resistance, isolation, and ability to function effectively in intense environmental situations [4]. The small size of these sensors enables them to be securely utilized over extended distances with minimized future maintenance operations [5]. One of the crucial benefits of fiber optics is that the same sensor can carry dual purposes. It can act in the same way as the sensing element for measuring the critical physical parameters and as a transmission medium for the detected signal [6].

Generally, distributed fiber-optic sensing (DFOS) can be classified into three types: (a) distributed temperature sensing (DTS), (b) distributed acoustic sensing (DAS), and (c) distributed strain sensing (DSS) [7]. DAS employs an optical time-domain reflectometer (OTDR) system to record high-frequency strain rates over long distances. The sensing range along the fiber length of a DAS system can be more than 10 km, with a spatial resolution less than 1 m and a temporal sampling frequency up to 10 kHz [8]. Since a well-protected sensing fiber can endure harsh environments, DAS is desirable in wellbore diagnostics to examine several aspects. It can serve as a noise log to detect leakage or estimate injection rate—e.g., [9,10], supporting the differentiation between the channeling, borehole flow, matrix flow, and fracture flow [11]. It can also be used for hydraulic fracture monitoring [12] and seismic acquisition [13]. DFOS is commonly used and has many applications in engineering and geophysics disciplines.

van der Horst et al. (2013) [14] reported using DAS for flow monitoring for tight gas well producers. The acoustic noise was monitored, and the production profiles were generated by the gas phase flowing from the perforations, which is then converted to flowrates using the DAS signal in a specified frequency band. This method has provided some quantitative production measurement but does not apply to oil well producers. A study shows the flow velocity estimation using low-frequency DAS signals for tracking the wellbore thermal slugging. Jin et al. (2019) [15,16] demonstrate the use of a combination of DTS and DAS for production monitoring. The method is used for oil producers with low producing rates where the DTS was utilized to measure steady-state borehole temperatures, and the DAS is used to measure transient borehole flow velocities by tracking the signals generated by thermal slugging. This research was further developed for liquid and gas two-phase flow, specifically for gas–oil two-phase slug flow [17,18,19]. The experiments’ findings using a vertical flow loop indicated that the acoustic and thermal signals measured by DAS are sensitive to low air injection. Also, the thermal signatures obtained from the DAS data mainly respond to the water phase in the flow, while the air phase provides unique characteristics in the acoustic domain.

Another study shows that DAS measurements for two-phase gas–liquid slug flow are used to estimate the velocity and extract velocity patterns. The research includes two methods, k-f transform, and distributed cross-correlation. The results are validated by a commercial conductance-based phasemeter [20]. Many DFOS data processing are based on qualitative approaches only. In this article, experiments are conducted to develop a quantitative analysis for multiphase flow in pipes using DAS, which can expand the applications of DFOS data and contribute to better multiphase flow characterization.

**Figure 1:**
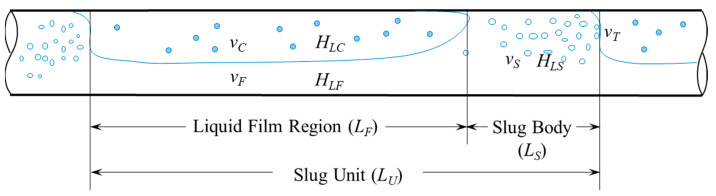
Physical structure of slug flow with its characteristic parameters.

Flow patterns in horizontal two-phase flows can range from dispersed, stratified, intermittent, and annular flow patterns depending on the rates of the phases, fluid properties, and pipe diameter [21]. Slug flow is a type of intermittent flow pattern, in which the gas and liquid phases flow alternatively. It is also one of the most common flow patterns encountered in the oil and gas transportation system [22]. This type of flow pattern have been extensively studied in the laboratory [23], such as [24,25,26,27], to name a few. The authors of [21,28] provide a detailed review of this flow pattern and the relevant modeling studies. A typical slug unit consists of a slug body region and a film region (also called gas pocket region), as depicted in Figure 1. Some previous studies claimed that the slug region could be considered as a dispersed bubble flow, and the film region as segregated flow [29]. The entire slug unit is moving forward at the translational velocity (*v_T_*), which is also the slug front velocity. Figure 1 illustrates the typical characteristic parameters for slug flow, including the translation velocity. From Figure 1, *L_U_* is the slug unit length, *L_S_* is the liquid slug body length, *L_f_* is the length of the liquid film region, vs. is the average mixture velocity inside the slug body, *v_F_* is the average liquid velocity of the liquid film region, and *v_C_* is the velocity of the gas pocket in the liquid film region.

It is essential to characterize the slug flow, not only because of its common occurrence in the oil and gas transportation system, but also due to its alternating behavior of liquid slugs and gas pockets which could pose a risk to the production system and compromise the safety of the pipelines, especially to the joints and elbows [30,31,32]. By monitoring the slug flow characteristics, such as frequency, translational velocity, and the lengths of the slug body and the liquid film regions, we will be able to assess their risks to the pipeline systems and the piping components, react immediately if the slug flow behavior is determined to be damageable, and assess the multiphase flow rates for individual pipelines.

In this research, low-frequency strain-rate analysis of the DAS is used to detect gas–oil two-phase slug flow and quantify its characteristics. Novel algorithms were developed to characterize the slug translational velocity, and the lengths of slug body and the liquid film regions that had never been discussed in previous studies. These parameters are essential for slug-induced risk assessment and flow rate estimation. This study also employed a high-speed camera next to the fiber cables for validation purposes, achieving a good correlation among the measurements under all conditions tested.

## 2. Materials and Methods

**Figure 2:**
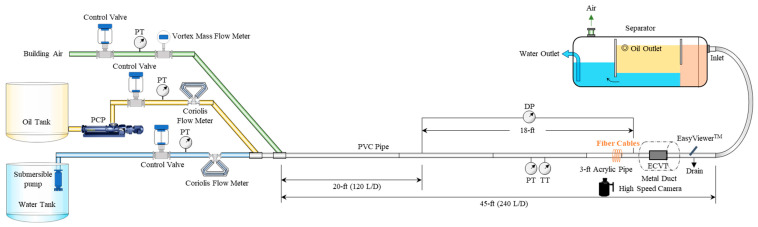
The schematic of the three-phase multiphase flow loop.

**Figure 3:**
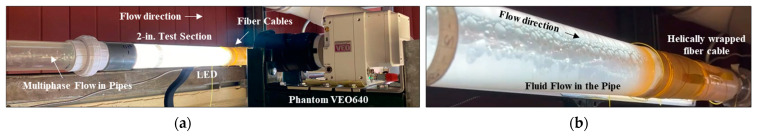
(**a**) Pictures of the testing section with the helically wrapped yellow fiber cables, and the high-speed camera taking videos. (**b**) A closer look at the wrapped fiber cable.

The two-phase gas–oil experiments were conducted in a three-phase flow loop at the Colorado School of Mines, consisting of a transparent polyvinyl chloride (PVC) horizontal test section measuring 14 m long, a 5.25 cm inner diameter, and a 6.03 cm outer diameter. Figure 2 shows the schematic of the facility. Figure 3 is a picture of the test section, high-speed camera, and the pipe helically wrapped with yellow fiber cable. A fiber cable was wrapped helically on a 35 cm long section and was connected to a Terra15 Treble DAS interrogator. The winding pitch is 0.112 cm. The data were acquired with a sampling rate of 10 kHz and a special sampling of 0.816 m along the fiber cable. The gauge length that was applied is 2.4 m. The flow loop had a differential pressure transducer to measure the pressure drop in the horizontal test section. Pressure and temperature transducers were installed at the test section to monitor the pressure and temperature during the tests. The sensors were all connected to a data acquisition system and recorded at a frequency of 10 Hz.

Mineral oil and compressed air were used as testing fluids in this study. The mineral oil had a density of 810 kg/m^3^ and a viscosity of 0.012 Pa∙s at 19 °C and atmospheric pressure. While the air had a density of 1.2 kg/m^3^ and a viscosity of 1.83 × 10^−5^ Pa∙s at 19 °C and atmospheric pressure. The oil was introduced to the system through a wye inlet connector, and then the building air from a compressor was introduced afterward. Various rates for the gas and liquid phases were examined by varying the control valve for each phase or the pump motor through the variable frequency drive (VFD). The superficial oil velocity (defined as the volumetric flow rate divided by the pipe cross-sectional area) varied from 0.2 to 0.8 m/s, while the gas phase varied from 0.16 to 0.78 m/s. The test matrix presented in this work is shown in Table 1.

The facility was equipped with a high-speed camera (Phantom VEO640, Wayne, NJ, USA) to acquire a side view of the fluid flow behavior in the acrylic test section (Figure 3a). The video was recorded at 200 Hz with a resolution of 1024 × 700. A light source was fixed behind the pipe to provide illumination. The camera was used to record the flow pattern of testing fluids that were circulated through the pipe. Camera videos were also processed and analyzed to obtain the slug flow characteristic parameters and used to validate the ones obtained from DAS.

## 3. Data Processing Workflow and Results

In this section, the data processing is discussed in two sections based on measurement technology. The first part, which is also the main one, describes the automated workflow developed in this study to detect and track the slugging signals in the gas–oil two-phase slug flow and obtain the slug flow characteristic parameters from the DAS data. It also includes a discussion of the workflow process and its accuracy. The second part shows the high-speed camera data processing of the tested cases and the slug parameters obtained. The comparison of the results from DAS and the high-speed camera is discussed afterward, followed by a subsection showing the slug flow variations with the flowing conditions.

### 3.1. DAS Data Processing Workflow

This workflow uses low-frequency strain-rate analysis of the DAS signal to demonstrate its capabilities of quantifying two-phase slug flow characteristic parameters, including the slug frequency, slug body length, slug translational velocity, slug unit, and the liquid film region lengths. The DAS can capture these signals due to the sensitivity of helically wrapped fiber to the slight pipe diameter change that is caused by the pressure differential exerted by a passing slug.

**Figure 4:**
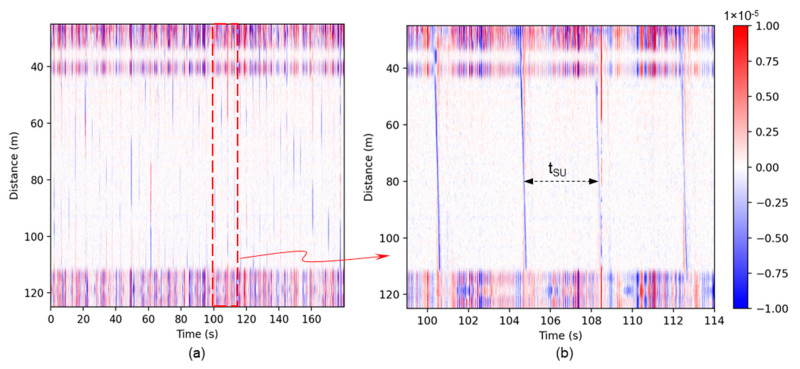
(**a**) Low-frequency DAS signal for slug flow; (**b**) fifteen-second time selected to illustrate the passing slugs in the signal.

The raw DAS data are read into the workflow for the selected beginning and ending times. To reduce noises and enhance the analysis, the median value is computed and removed from the channel direction, and then a low-pass filter is used to filter the data, displaying signals that are lower than 20 Hz. Figure 4a shows the low-frequency data for gas–oil two-phase slug flow. The horizontal axis is time, while the vertical axis shows the channel number, which corresponds to the fiber length from the interrogator. The wrapped test section is from around 45 m to 110 m. A zoomed portion of the data over a fifteen-second period in Figure 4b, presented four slugs passing which are indicated by the red and blue inclined lines. *t_SU_* denotes the time interval between the two adjacent slugs at a position in the test section around 80 m in fiber length from the interrogator (near the middle of the fiber cable wrapped section).

**Figure 5:**
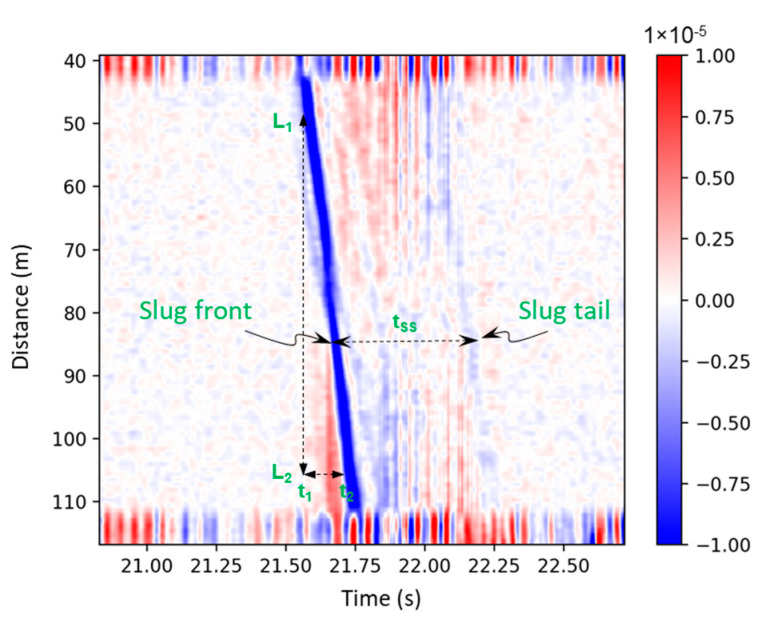
DAS signal of a single slug in the gas–oil two-phase slug flow.

Figure 5 is a visualization of the DAS signal of a single slug passing through and the identification of the slug front and tail. One way to obtain the slug translational velocity, *v_TB_*, is to calculate the slope of the blue line, which indicates the slug front, given in Equation (1):

(1) $$v_{TB}=\frac{\emptyset \left(L_{2}-L_{1}\right)}{t_{2}-t_{1}}\;,\;$$

where (*L*_2_ − *L*_1_) represents the distance between two points shown in the DAS data; ∅ is a correction factor that converts the fiber cable distance to the actual distance in the test section; (*t*_2_ − *t*_1_) represents the time required for this slug traveling from *L*_1_ to *L*_2_.

*t_SS_* in Figure 5 is the time interval between slug front and tail, $\left(t_{SlugTail}-t_{SlugFront}\right)$, representing the time required for the slug body to pass a particular point on the test section. It can be used to determine the slug body length, given in Equation (2):

(2) $$L_{S}=v_{TB}t_{SS}=v_{TB}\left(t_{SlugTail}-t_{SlugFront}\right)\;,$$

The slug unit length, *L_U_*, can be determined using Equation (3), where *t_SU_* is the time interval between two adjacent slugs (Figure 4b):

(3) $$L_{U}=v_{TB}t_{SU}\;,$$

The length of the film region, *L_F_*, are then calculated using Equations (4):

(4) $$L_{F}=L_{U}-L_{S}\;,$$

In this paper, we propose a more automatic workflow for the determination of the translational velocity and the lengths of the slug unit, slug body, and the film region. The following paragraphs describe this workflow.

**Figure 6:**
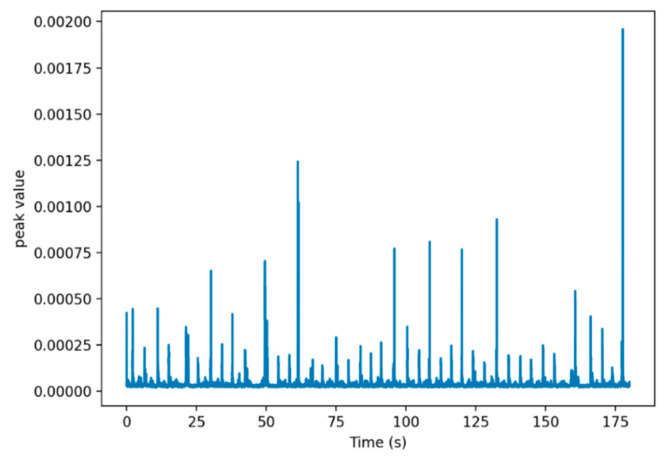
The peak value of the slugs over a three-minute recorded time for the case of 0.2 m/s oil velocity and 0.16 m/s gas velocity.

We selected the data between channels 45 to 110 in the data processing to eliminate the excessive noise beyond this range. These channels are associated with the wrapped fiber section near the camera section. A sum is then taken in the channel direction after taking the absolute value of the signal and plotted against the time to identify the time of each slug, which are presented as the peaks in Figure 6. Each of the slugs was given a unique slug index number (ID#) to be used in comparison with the camera data at later stages.

The peaks in Figure 6 were counted to determine the slug frequency, *f_S_*, which is the number of slugs divided by the recording time. Note that the peaks that are very close are counted as one peak, which should correspond to a single slug but probably with a more complex structure. This prevents the overcounting of slugs due to adjacent peaks when calculating the slug frequency.

**Figure 7:**
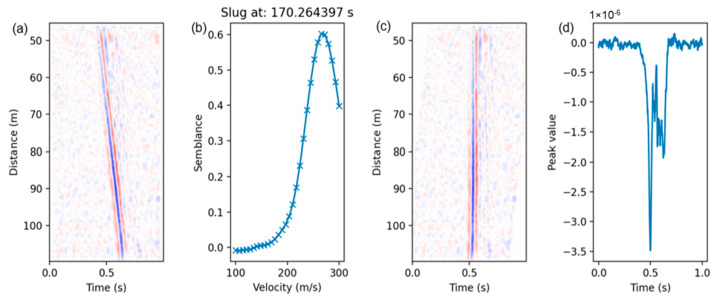
An example of quality check plots for workflow evaluation at slug#45. (**a**) Slug waterfall plot; (**b**) semblance as a function of testing velocity; (**c**) waterfall plot after applying linear moveout using velocity that gives the highest semblance value in (**b**); (**d**) vertical stacking in the distance direction.

To determine the slug translational velocity, we used semblance, a quantitative measure of the waveform similarity that estimates the consistency of the waveforms from different channels. The process is explained in Figure 7, which includes four plots: (a) slug waterfall plot, (b) semblance vs. velocity, (c) waterfall plot after applying linear moveout using velocity that gives the highest semblance value from the previous plot, (d) vertical stacking in the distance direction to determine the width of the negative peak for slug body length calculation.

The plot generation processes are described as follows:

(a) The waterfall, a plot of distance versus time, is generated for each slug.
(b) A linear moveout correction (*LMO*) is applied, which is a velocity correction to shift traces in time based on an assumed velocity. The best velocity reflects the most consistency of the waveform among traces in time after the correction. The following equation represents the linear moveout correction:
  (5) $$t_{LMO}\left(n,v\right)=\frac{d\left(n\right)}{v}\;,$$
  where *n* represents the trace number in the channel direction, *d*(*n*) is the fiber distance of the channel n, *v* represents the testing velocity, and $t_{LMO}$ represents the time shift applied to the channel.
(c) The semblance is a quantitative measure of the waveform similarity from different channels, which is a metric commonly used in seismic processing. It is calculated using the following equations:
  (6) $$S=\sum_{t=t_{0}-∆t}^{t=t_{0}+∆t}{\left(\sum_{n=1}^{N}f_{n}\left(t-t_{LMO}\left(n,v\right)\right)\right)}^{2},$$
  where S is the summation of all waveforms in the channel direction, which is then squared and summed again in the time direction. *f_n_*(*t*) represents the data value of channel n at time t. N is the total number of channels, Δt is the half window length in time for the semblance calculation, and $t_{0}$ is the center of the time window.
  (7) $$E=\sum_{t=t_{0}-∆t}^{t=t_{0}+∆t}\sum_{n=1}^{N}{f_{n}}^{2}(t-t_{LMO}\left(n,v\right))\;,$$
  where *E*(*t_*0*_*) is the summation of the square of all waveforms in both channel and time directions.
  (8) $$R\left(v\right)=\frac{S-E}{\left(N-1\right)\;E}\;,$$
  Finally, R is the normalized semblance value of all the channels for the testing velocity v. An array of the different testing velocities versus semblance can be obtained, and the best velocity is chosen at the maximum semblance value which represents the highest consistency of waveforms after linear moveout correction. This can be used as the estimation of the slug translational velocity after applying a fiber-length-to-pipe-length ratio of 169:1.
(d) All the signals are stacked vertically in the channel direction after the linear moveout correction using the best velocity, and the negative peak is identified with its start and end times, which represent the slug’s front and tail.

The slug characteristic parameters, including the slug body length, slug unit length, and the length of the film region, are calculated using Equations (2)–(4), respectively. An example of one of the slugs with its calculated parameters from the process in this section is shown in Table 2.

**Figure 8:**
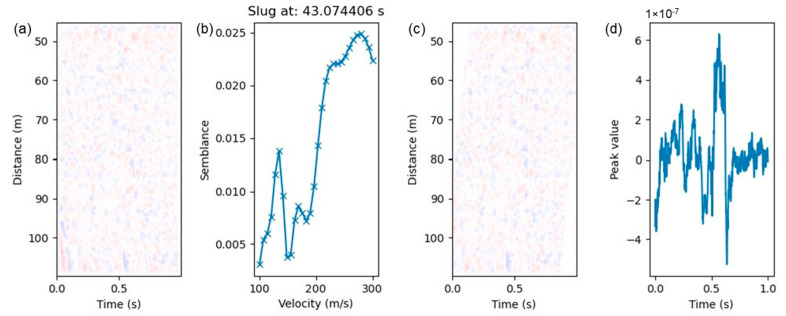
An example of a bad slug (ID #12) that does not give good quantitative analysis. (**a**) Slug waterfall plot; (**b**) semblance as a function of testing velocity; (**c**) waterfall plot after applying linear moveout using velocity that gives the highest semblance value in (**b**); (**d**) vertical stacking in the distance direction.

To increase the accuracy of the quantitative analysis on the slug characteristic parameters, a threshold for the calculated semblance after correction is selected at 0.1 to filter out some of the bad signals that may not represent an actual or complete slug signal and do not give a good quantitative analysis. An example is shown in Figure 8, where the automated workflow does not predict a reliable measurement for slug#12. This is also observed in Table 3 where the semblance after correction is very low (highlighted in red). In this example, the DAS signal in the selected window only contains a little part of a slug body shown at the bottom left in the waterfall plot (Figure 8a), resulting in unreliable quantification of the slug characteristics as anticipated. Nonetheless, these “bad” slugs represent a low percentage of the total population of the detected slugs that have reliable quantitative analysis and velocity measurement. Table 4 summarizes the percentage of the slugs with reliable quantitative analysis. The characteristic parameters of the slug were analyzed for these slugs and evaluated with data from the high-speed camera. The comparison is discussed in the later sub-section.

**Figure 9:**
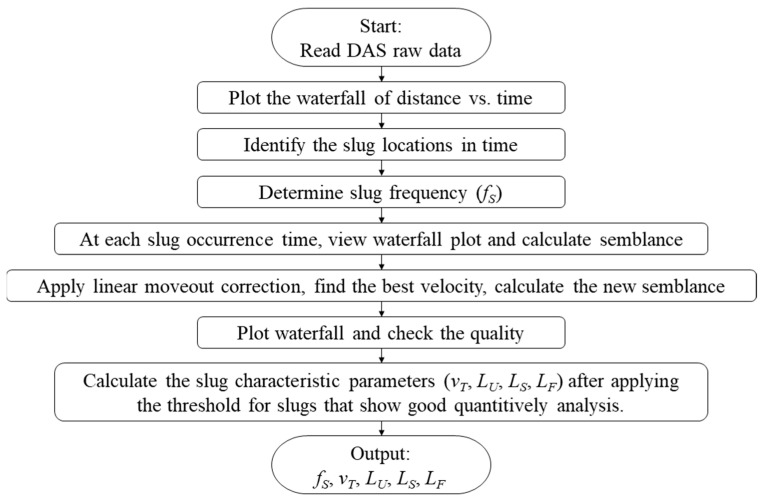
Workflow of the DAS data processing for slug flow characterization.

The summary of the workflow steps in the DAS data processing and the slug characteristic parameters that can be obtained is presented in Figure 9.

### 3.2. High-Speed Camera Data Processing

**Figure 10:**
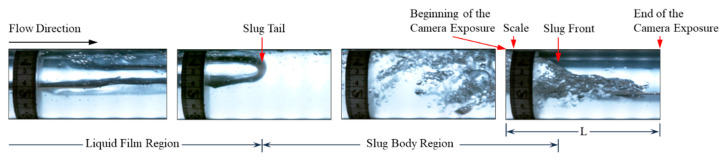
Slug flow captured from a recorded video by a high-speed camera at 0.16 m/s gas velocity and 0.2 m/s oil velocity.

The camera data processing involves the recorded videos from the high-speed camera of the flow at each of the gas–oil two-phase flow cases presented in Table 1 previously. Each video is recorded after reaching the stabilization of the flow for about three minutes at a frequency of 200 Hz. Figure 10 presents a capture of a slug passing by for a liquid superficial velocity at 0.2 m/s and gas superficial velocity at 0.16 m/s. A scale was attached to the pipe which was used as a point of entry for the slug’s front and tail. The videos are then analyzed entirely and each slug that is passing would have a unique slug index and the following data processing for it:

(a) The time when the slug front starts is determined once reaching the scale, *t_SF_*.
(b) The time when the slug tail reaches the scale is documented, *t_ST_*.
(c) The time when the slug front reaches the beginning of the camera exposure is recorded, *t_SF_In_*.
(d) The time when the slug front reaches the end of the camera exposure is recorded, *t_SF_Out_*.
(e) Translational velocity, *v_T_*, is obtained by finding the length of the horizontal section that is exposed to the camera divided by the duration of exposure of each slug, i.e., *v_T_ = L/*(*t_SF_Out_ − t_SF_In_*).
(f) Slug unit length is determined by *L_U_ = v_T_* (*t_SF_*_1_ *− t_SF_*_2_), where (*t_SF_*_1_ *− t_SF_*_2_) is the time interval between two adjacent slugs.
(g) The length of the slug body is determined by *L_S_ = v_T_* (*t_SF_ − t_ST_*), and the length of the film region is determined using Equation 4 as presented previously.
(h) Slug frequency, *f_S_*, is determined by counting the number of slugs divided by the corresponding recording time.
(i) For each of the slug characteristic parameters obtained, the average and the median values were calculated over the full three-minute duration of the recorded video.

### 3.3. Data Validation

**Figure 11:**
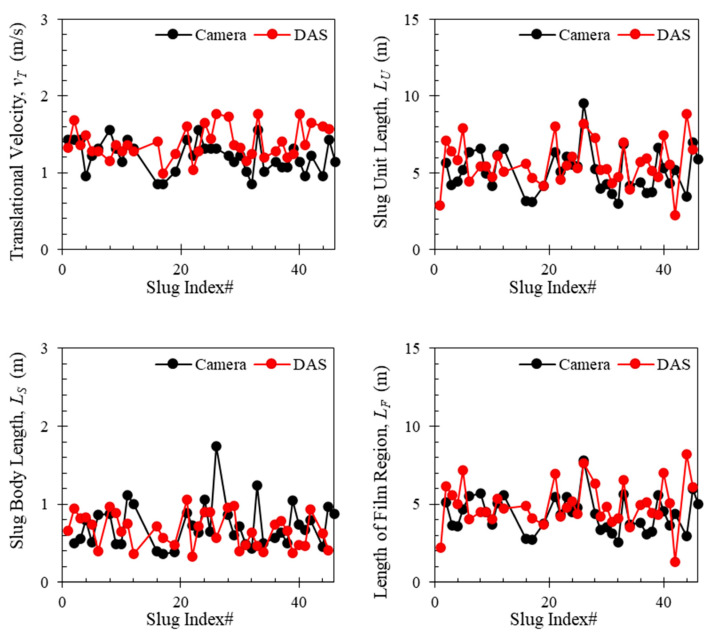
Comparison between the slug characteristic parameters from the DAS data and the camera for the case of 0.2 m/s oil superficial velocity and 0.16 m/s gas superficial velocity.

The accuracy of the DAS workflow process is validated using the high-speed camera videos which serve as the ground truth. An example of the comparison is presented in Figure 11, which shows the comparison of *v_T_*, *L_U_*, *L_S_*, and *L_F_*, between the data obtained from DAS (red points) and the high-speed camera videos (black points). The oil superficial velocity is 0.2 m/s, and the gas superficial velocity is 0.16 m/s. A good match between the DAS data and the high-speed camera data is observed.

One might have noticed that the slug characteristics are not the same at the same flowing condition. This fluctuation is the nature of slug flow. Dozens of previous studies have tried to develop statistical models using laboratory data to predict the slug characteristics, especially the slug length which is a critical parameter for facility design [33,34,35]. With the development of DAS, we can now monitor the slug characteristics directly in the field in real time.

**Figure 12:**
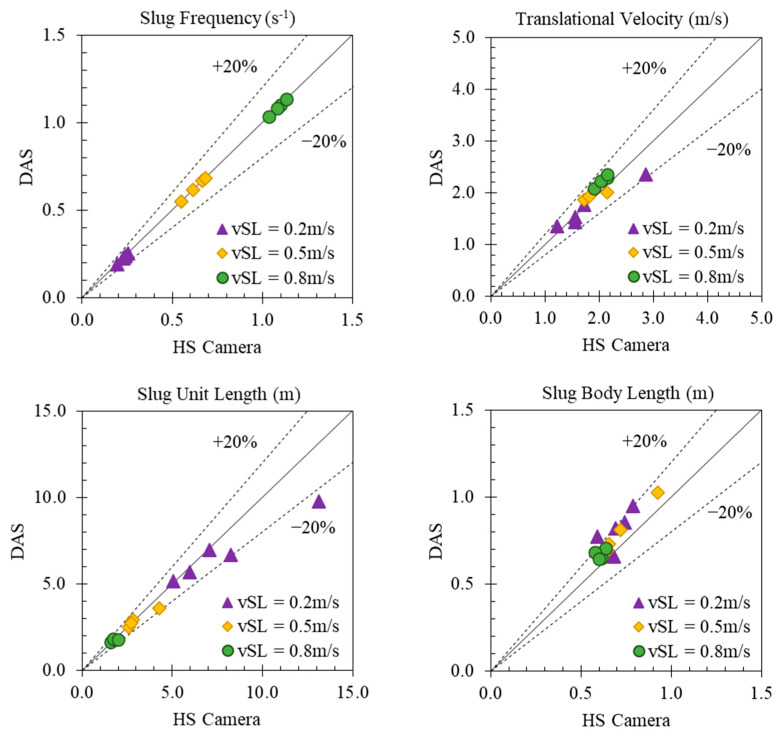
Comparison between the median value from DAS and high-speed camera.

**Figure 13:**
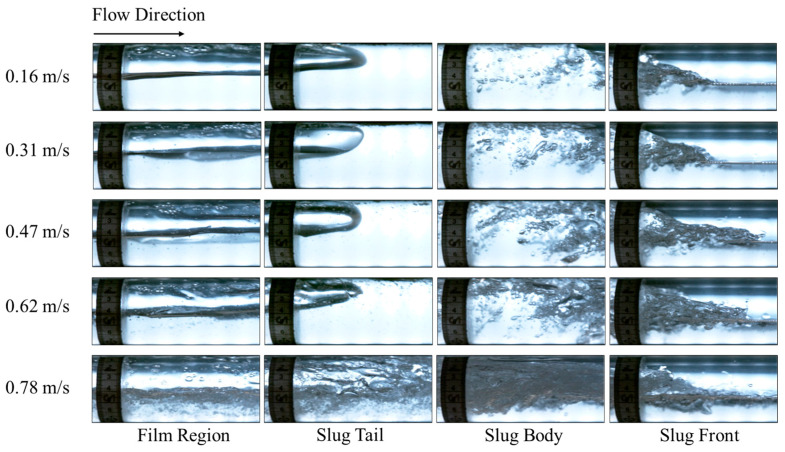
Flow pattern captured from the high-speed camera at 0.2 m/s oil superficial velocity and different gas flow rates.

To further evaluate the performance of DAS and the data processing workflow, we generated cross plots for all the gas–oil two-phase experimental data points, and the comparison of the median values is shown in Figure 12. Considering the median value of each slug parameter in the 13 experiments over the three-minute period of recorded data, a very good match can be observed with the camera data with an error bar of less than 20% in the majority of the cases. One astonishing observation is that the DAS can detect the number of slugs or quantify the slug frequency, very successfully for the conditions investigated in the current study. For the other parameters, the error seems to increase with the increase in gas flow rate or decrease in liquid flow rate. One of the reasons could be the reduced number of slugs investigated as the gas flow rate increases or the liquid flow rate decreases, so a smaller number of measurements are averaged. Another reason could be the change in the physical properties of the slug body, i.e., more gas is present in the slug body as the gas flow rate increases or liquid flow rate decreases, and the liquid slugs become more chaotic, frothy, and shorter, making the signal less clear and more difficult to be processed [31,32,36]. This phenomenon is illustrated in the images from the high-speed camera in Figure 13. On the other hand, we also assessed the measurement uncertainty from the high-speed camera. The errors for translational velocity, slug unit, and body lengths range from 0.87% to 1.1%. These errors are not visible in Figure 12.

### 3.4. Slugs at Different Flowing Conditions

**Figure 14:**
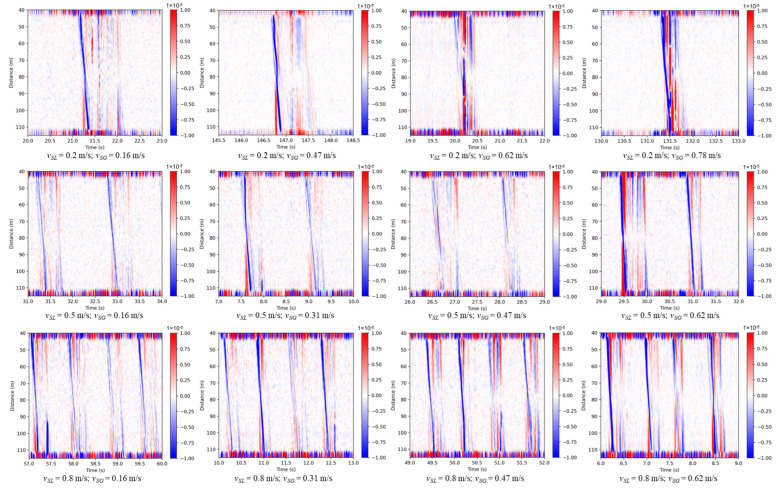
DAS signals at different flowing conditions (*v_SL_*: liquid superficial velocity, *v_SG_*: gas superficial velocity. Superficial velocity is defined as the volumetric flow rate divided by the pipe cross-sectional area).

In this subsection, we will show the slug flow behavior at different flowing conditions. Figure 14 shows the DAS signal after applying the low-pass filter and the median value removal in the channel direction, for three different liquid flow rates and four gas flow rates. The time duration is 3 s for all the figures. It can be clearly noticed that the slug frequency increases as the liquid flow rate increases. Moreover, the lines become steeper as the gas or liquid velocity increases, indicating an increase in the slug translational velocity. This observation is consistent with other previous studies for gas–liquid flow in horizontal pipes [37]. The slug body length cannot be directly read from the plots, as it is a function of time duration and also the slug translational velocity.

**Figure 15:**
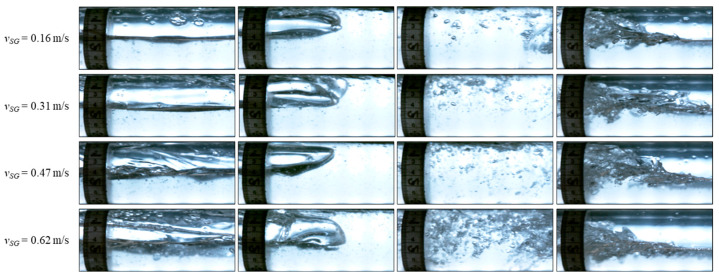
Pictures from high-speed camera videos for the slugs shown in Figure 14 for *v_SL_* = 0.5 m/s.

**Figure 16:**
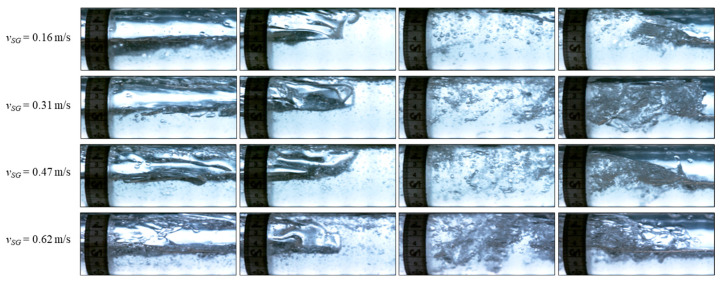
Pictures from high-speed camera videos for the slugs shown in Figure 14 for *v_SL_* = 0.8 m/s.

The pictures of some slugs in Figure 14 are shown in Figure 15 and Figure 16 for 0.5 m/s and 0.8 m/s liquid superficial velocities, respectively. As the gas velocity increases, more gas bubbles are entrained inside the slug body due to the high turbulence in the mixing zone at the slug front [38]. This increases the noise in the DAS signal, as depicted in Figure 14. We suspect that the intensity of the DAS signal within the slug body may correlate with the turbulence and entrained gas bubbles within the slug body. Further investigations are necessary to better understand this relationship quantitatively.

## 4. Discussion

Slug flow characterization is of great importance to many field applications. In the oil and gas industry, knowing the slug flow characteristics, such as the slug body length, translational velocity, and frequency, is crucial for facility design and operation. For example, the size and internal design of the separator should be able to handle the slugs to avoid flooding or ineffective separation. Slug catcher design also requires information on the slug flow characteristic parameters to achieve optimal gas–liquid separation [39]. Topside slugging control for a riser system requires real-time slug flow monitoring that is automated with the control valve systems [40]. Moreover, slug flow can accelerate corrosion and erosion due to their high translational velocities and the highly turbulent zoom at the slug front that could possibly lead to cavitation [41,42]. Knowing the slug flow characteristics, particularly in real time, empowers us to more confidently assess the risk of slug-flow-induced or -accelerated corrosion and erosion and adjust the operating parameters accordingly to minimize the risks. The current most widely used method in the field for multiphase surface facilities design and flow assurance is based on predictive models that are developed based on laboratory data or very limited field data. Their accuracy becomes questionable as it is scaled up to the field application. Because the slug flow behavior is transient and dramatically diverse in both the spatial and time domains, even at the same flowing conditions [22,43], real-time monitoring in the field will empower us to more effectively track their behaviors, allowing us to better optimize facility design and operation.

DAS is advanced in many aspects compared with other point sensors, such as γ-ray sensors, wire-mesh sensors, electrical impedance (capacitance and conductance) sensors, microwave sensors, optical sensors, etc., as previously mentioned in the introduction. It is completely non-intrusive and does not disturb the existing facility. It can provide measurements at multiple different locations and over a long distance in real time, instead of a point measurement like the other types of sensors, making it more cost-effective. Another big advantage is that DAS can work in harsh environments like downhole, where the other type of sensor can hardly survive.

In this paper, we introduce a new method for characterizing the slug translational velocity using semblance, showing great alignment with the observations from the high-speed camera that serve as the ground truth. Moreover, we demonstrated a new algorithm to characterize lengths of slug body, slug unit, and the liquid film region, which are the most critical parameters required in field design and operation as discussed above. The algorithm requires the fiber to be densely wrapped around the pipe, which increases the sensitivity of the strain rate measurement due to the pipe diameter variations caused by the slug-induced pressure fluctuations (see Figure 5).

The slug flow behavior should be related to different types of fluids and compositions, and the DAS signal should respond differently depending on the fluid properties and the slug flow behaviors. In the next phase, we are going analyze the DAS signals for other different fluid compositions, including gas–water, oil–water, and gas–oil–water flows.

Furthermore, we will investigate the causes of the close peaks in Figure 6. There is a possibility that these close peaks are induced by large gas pockets within long slug bodies, but we will further investigate this phenomenon from both fluid flow dynamics and data processing perspectives. Frequency domain dynamic averaging (FDDA) and/or activation function dynamic averaging (AFDA) methods will be tested.

## 5. Conclusions

A series of experiments to investigate the capability of DAS to quantify the slug characteristics were conducted in a horizontal pipe under different flowing conditions. A low pass filter and median value removal were applied to better extract the slug signals. We introduce a new method for characterizing the slug translational velocity using semblance, showing great alignment with the observations from the high-speed camera that serve as the ground truth. Moreover, we demonstrated a new algorithm to characterize the lengths of the slug body, slug unit, and the liquid film region. The algorithm requires the fiber to be densely wrapped around the pipe, which increases the sensitivity of the strain rate measurement due to the pipe diameter variations caused by the slug-induced pressure fluctuations.

Real-time slug flow monitoring and characterization are crucial for many industries that involve slug slow, such as petroleum engineering, geothermal wells that have two-phase flow production, nuclear engineering, etc. This research demonstrates the capabilities of DAS to quantitatively measure the slug flow characteristic parameters, especially the lengths of the slug body, slug unit, and the liquid film region, which are the most critical parameters required in field facility design and operation. This technology will offer the industry a more optimal solution for facility design and ensure safer operational practices.

## Figures and Tables

**Table 1:** Test matrix in the current study.

Case# ^1^	Superficial Oil Velocity [m/s]	Superficial Gas Velocity [m/s]
1–5	0.2	0.16, 0.31, 0.47, 0.62, 0.78
6–9	0.5	0.16, 0.31, 0.47, 0.62
10–13	0.8	0.16, 0.31, 0.47, 0.62

^1^ The case number # in each row corresponds to different superficial gas velocity. For example, Case#1 corresponds to an 0.2 m/s superficial oil velocity and 0.16 m/s superficial gas velocity; Case#3 corresponds to an 0.2 m/s superficial oil velocity and 0.47 m/s superficial gas velocity.

**Table 2:** Characteristic parameters for slug#45 determined from the DAS signals using the proposed approach.

Parameters	Value	Parameters	Value
Slug#ID	45	*t_SlugFront_* (s)	0.406
Slug occurrence time (s)	170.26	*t_Tail_* (s)	0.668
Semblance before correction	−0.008588	*t_SS_* (s)	0.262
Best velocity (m/s)	265.52	Slug Frequency (s^−1^)	0.239
Semblance after correction	0.602624	Slug Body Length, *L_S_* (m)	0.410
Slug translational velocity, *v_T_* (m/s)	1.565	Liquid Film Region Length, *L_F_* (m)	6.125
*t_SU_* (s)	4.176	Negative Peak Time (s)	0.500
Slug Unit Length, *L_U_* (m)	6.536	Negative Peak Value	−3.487 × 10^−6^

**Table 3:** Characteristic parameters for slug#12 determined from the DAS signals using the proposed approach that does not give good quantitative analysis.

Parameters	Value	Parameters	Value
Slug#ID	412	*t_SlugFront_* (s)	0.000
Slug occurrence time (s)	43.074	*t_Tail_* (s)	0.673
Semblance before correction	−0.004933	*t_SS_* (s)	0.673
Best velocity (m/s)	279.31	Slug Frequency (s^−1^)	1.429
Semblance after correction	0.024887	Slug Body Length, *L_S_* (m)	1.108
Slug translational velocity, *v_T_* (m/s)	1.6464	Liquid Film Region Length, *L_F_* (m)	0.046
*t_SU_* (s)	0.7	Negative Peak Time (s)	0.045
Slug Unit Length, *L_U_* (m)	1.154	Negative Peak Value	0.363 × 10^−6^

**Table 4:** Percentage of the slugs with reliable quantitative analysis from the DAS automated algorithm.

Case #	*v_SO_* [m/s]	v_SG_ [m/s]	P * [%]
1	0.2	0.16	78
2		0.31	84
3		0.47	88
4		0.62	73
5		0.35	63
6	0.5	0.16	81
7		0.31	90
8		0.47	88
9		0.62	94
10	0.8	0.16	94
11		0.31	94
12		0.47	95
13		0.62	98

* P: Percentage of slugs with reliable quantitative analysis and used for slug characteristic parameter determination.

## Data Availability

Data may be provided upon request.
